# Water and nitrogen in-situ imaging detection in live corn leaves using near-infrared camera and interference filter

**DOI:** 10.1186/s13007-021-00815-5

**Published:** 2021-11-13

**Authors:** Ning Zhang, Peng-cheng Li, Hubin Liu, Tian-cheng Huang, Han Liu, Yu Kong, Zhi-cheng Dong, Yu-hui Yuan, Long-lian Zhao, Jun-hui Li

**Affiliations:** College of Information and Electrical Engineering, China Agricultural University, Beijing, 100083 China

**Keywords:** Corn leaf, Multispectral imaging, Near infrared camera, Near-infrared, Nitrogen content, Water content

## Abstract

**Background:**

Realizing imaging detection of water and nitrogen content in different regions of plant leaves in-site and real-time can provide an efficient new technology for determining crop drought resistance and nutrient regulation mechanisms, or for use in precision agriculture. Near-infrared imaging is the preferred technology for in-situ real-time detection owing to its non-destructive nature; moreover, it provides rich information. However, the use of hyperspectral imaging technology is limited as it is difficult to use it in field because of its high weight and power.

**Results:**

We developed a smart imaging device using a near-infrared camera and an interference filter; it has a low weight, requires low power, and has a multi-wavelength resolution. The characteristic wavelengths of the filter that realize leaf moisture measurement are 1150 and 1400 nm, respectively, the characteristic wavelength of the filter that realizes nitrogen measurement is 1500 nm, and all filter bandwidths are 25 nm. The prediction result of the average leaf water content model obtained with the device was R^2^ = 0.930, RMSE = 1.030%; the prediction result of the average nitrogen content model was R^2^ = 0.750, RMSE = 0.263 g.

**Conclusions:**

Using the average water and nitrogen content model, an image of distribution of water and nitrogen in different areas of corn leaf was obtained, and its distribution characteristics were consistent with the actual leaf conditions. The experimental materials used in this research were fresh leaves in the field, and the test was completed indoors. Further verification of applying the device and model to the field is underway.

## Background

Water and nitrogen are indispensable components for corn (*Zea mays*) growth and development. Water is essential for photosynthesis and is the main factor affecting metabolism [1]. Nitrogen is the main component of proteins and hence affects enzyme activity; it is also an essential element in the chlorophyll molecule. The nitrogen cycle plays a key role in the growth of corn, affecting morphology and yield [2]. The water content of corn leaves is the best indicator of the level of water profit and loss by corn [3], whereas corn nitrogen status is an important index of growth and yield of corn [4]. Achieving rapid real-time non-destructive testing of the water and nitrogen content in corn leaves has significance in research pertaining to leaf photosynthesis, diagnosing field crop growth, monitoring drought, and predicting crop yield [5].

Due to the electromagnetic absorption and scattering characteristics of the material, traditional spectroscopy technologies cannot be used for imaging detection in different areas, whereas near-infrared band technologies can be easily used for non-destructive testing. Hyperspectral imaging technology has been widely used in food [6], medicine [7], and crops [8] in recent years, owing to its fast and accurate characteristics. Applied research in crops mainly focuses on testing indicators of plant leaves, including water, nitrogen, chlorophyll, and other indicators [9]. Clevers et al*.* [10] collected data in the 970–1200 nm spectral region, which is sensitive to water absorption characteristics and can be used to accurately predict the water content in the plant canopy. Khan et al*.* [11] used hyperspectral analysis to analyze the effect of temperature on the nitrogen content in wheat leaves. The results showed that temperature has a negligible effect on the predicted value of nitrogen content. However, the use of hyperspectral instruments mainly focuses on obtaining the average value on the sample surface under laboratory conditions. Only a few studies have realized imaging analysis of the chemical composition of different sample surface areas in laboratory settings. There is limited literature detailing the realization of crop leaf water, nitrogen, and other chemical components in living organisms using in-situ imaging detection. The main reason for this is that a hyperspectral instrument with a high wavelength resolution and low luminous flux needs an external light source requiring high power consumption and a high intensity source to obtain the leaf spectrum data. Factors such as complex instrument accessories, large volume, heavy weight, and low luminous flux restrict the application of hyperspectral imaging. Hence, it is difficult to achieve in-situ imaging detection in the field using hyperspectral imaging.

Near-infrared cameras are characterized by a small size, small weight, high luminous flux, and high spatial resolution. Furthermore, the cameras have an adjustable aperture and a large-diameter lens, which allows online measurement without the need for an auxiliary light source [12]. However, general near-infrared cameras do not have wavelength resolution capability; these cameras need to have band-pass filters installed with different center wavelengths, thus becoming multispectral cameras with capability for a specific wavelength resolution. In the study of Bing Lu et al. [13], the imaging results of an ordinary camera with a filter and a hyperspectral instrument were compared. The results showed that the near-infrared camera, which was derived from a common camera combination filter, and the hyperspectral imaging instrument exhibited the same accuracy. Kobori and Tsuchikawa [14] obtained hyperspectral images at 1450 nm and achieved a high-precision prediction of leaf water content using the near-infrared camera with a filter, indicating that near-infrared chemical imaging technology can be used as a novel method to monitor plant physiological indicators. Widjaja Putra and Soni [15] evaluated vegetation index under different lighting using a red/green/blue (RGB) camera combination filter and cameras installed with a near-infrared red (NIR-R) and near-infrared red-edge (NIR-RE) band filter. The results showed that the overall performance was better for the camera installed with the NIR-RE frequency band filter.

In this study, we constructed a near-infrared imaging device, which is suitable for in-situ inspection of blades on site. Based on simulation analysis of the hyperspectral data of fresh corn leaves, the key parameters such as the characteristic wavelength position, quantity, bandwidth, and offset limit required for leaf nitrogen and leaf water measurement were obtained. According to the key parameters of the filter, bandpass filters with different center wavelengths were installed in front of the near-infrared spectrometer, and a near-infrared imaging device with wavelength resolution capability was obtained. In this study, multispectral imaging technology was used to determine the visual expression of water content and nitrogen content in maize leaves. We aimed to use near-infrared imaging to characterize the comprehensive index of drought resistance of crops, based on the average water content and the difference (WV, WM, DVM) and dynamic difference values (∆WV, ∆WM, ∆DVM) of leaf veins and mesophyll during the critical growth period.

## Methods

### Material selection and test

**Fig. 1:**
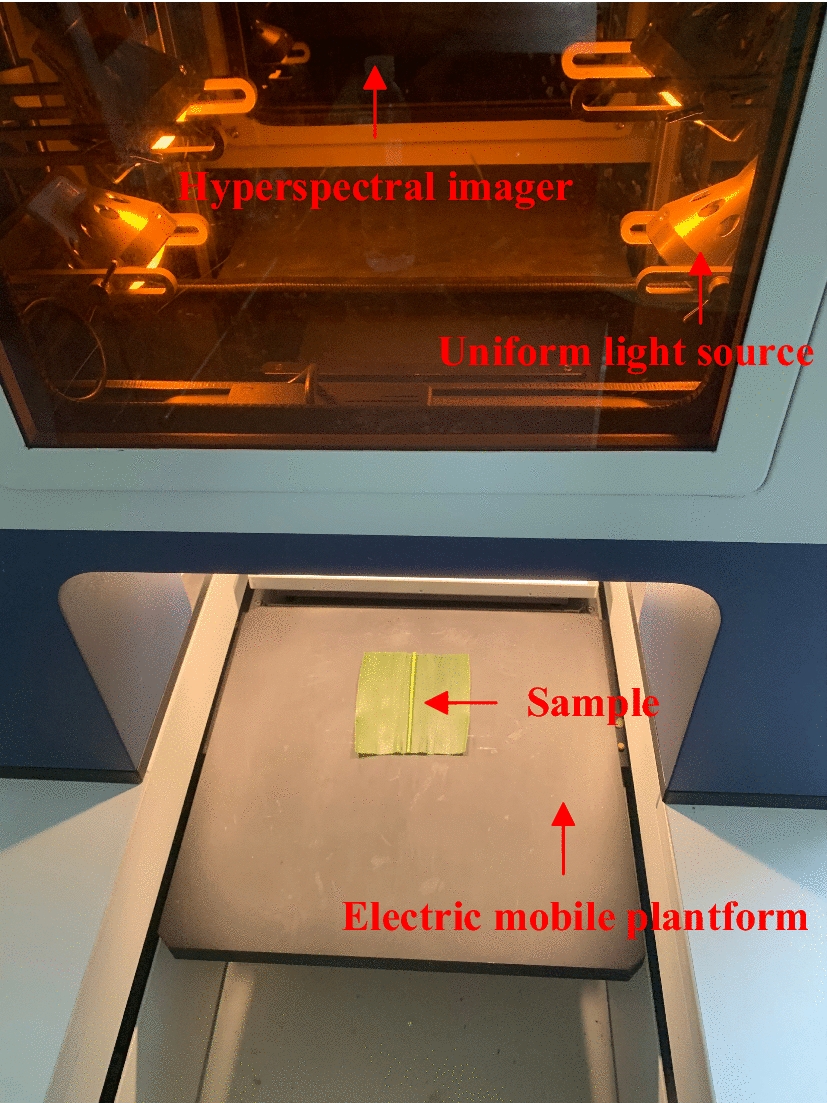
Gaia Sorter hyperspectrometer

We used a Gaia Sorter near-infrared hyperspectral instrument (Fig. 1). The performance configuration and working parameters of the instrument were as follows: a uniform light source composed of four bromine tungsten lamps, a 56-mm fixed-focus near-infrared camera lens, an N17E spectrometer, an AVT detector, and a computer box. The working principle of the hyper-spectrometer involves placing the experimental sample on an electronically controlled mobile platform and obtaining the hyperspectral cube information of the test sample using the push sweep method. We used an XEVA-0538 near-infrared camera (Xenith, Belgium) with a T2SL near-infrared focal plane array and InAs/GaSb type two superlattice detectors. The filter used was a traditional coated bandpass filter (Edmund, BURRINGTON, USA), with 1150 nm and 1400 nm center wavelengths, 25 nm bandwidth, 50 mm diameter, band frame, and optical density greater than 4.0. The near-infrared light source was a 100 W Phillips infrared bulb. In this experiment, the nitrogen analyzer was FOSS automatic Kjeltec TM 8400 (FOSS, Denmark). The accuracy of the electronic balance used in this experiment was 0.0001 g (Sartorius Instrument Systems Ltd., Beijing, China).

The experimental samples were collected from different test fields at the Shang Zhuang Experimental Station of Chinese Agricultural University. Corn leaves at different stages, including the seedling, jointing, heading, filling, and maturity stages, were selected. Leaves with a flat surface and intact mesophyll tissue were selected, stored in a fresh-keeping bag, and labeled. In total, 60 samples were collected.

After sampling, the samples were immediately sent to the hyperspectral laboratory of China Agricultural University for hyperspectral image acquisition and subsequent processing. First, the power was turned on and Spec View software (Spec View Ltd., Uckfield, UK) was run. After the system had warmed up for 30 min, a series of operations, such as focusing, were performed. The final determination of the motorized stage moving speed was 0.42 cm/s and the exposure time was 0.09 s. The distance between the sample and the hyperspectral camera was set to 30 cm, and the left and right, up and down, and illumination angles of the four lamps were adjusted to 45° and 75°, respectively.

The Kjeldahl method was used for measuring leaf nitrogen content. After image acquisition, the main leaf vein and the leaf mesophyll tissue were separated, their quality was measured, and then they were cut into pieces and mixed evenly for measurement. A uniformly mixed sample (0.6 g) was heated at 420 °C for 30 min and two copper sulfate (Zouping Tengchuang Biotechnology Co., Ltd., Shandong, China) and potassium sulfate (Zouping Tengchuang Biotechnology Co., Ltd.) catalytic tablets were added to accelerate digestion until the liquid was completely clear. After cooling, distillation titration was performed using the FOSS nitrogen analyzer. A blank control sample was tested before using the analyzer on samples.

The specific process and results of leaf water measurement have been mentioned in another article to be published in November 2021. Therefore, herein, we mainly introduce the key parameters for achieving leaf nitrogen measurement.

### Processing method

#### Hyperspectral image correction and data preprocessing

Considering the influence of factors, such as the dark current of the charge-coupled device camera, there is a need for correcting the collected hyperspectral data image [16]. The image captured when the camera lens is completely covered by the lens cover is the dark background value. The image captured when the hyper-spectrometer camera is aimed at a standard whiteboard is the standard whiteboard data. The correction formula is as follows:

1 $$A_{i} = - \lg \left( {\frac{S - B}{{W - B}}} \right)$$

where, *A*_*i*_ represents the corrected spectral absorbance data, *B* is the dark background data with a theoretical 0% reflectance, *W* is the standard white board data with a theoretical 99% reflectance, and* S* is the sample raw hyperspectral data.

ENVI 5.1 (ITT Visual Information Solutions, Boulder, UT, USA) was used to collect data from the region of interest of different mesomorphs and calculate the number of hyperspectral samples (60) × number of wavelengths (256) of the spectral data matrix for the average absorbance. The unprocessed raw spectral data include a large number of redundant signals and data with useful information; hence, it is difficult to conduct accurate data mining [17]. The spectral data should be preprocessed to eliminate the influence of spectral scattering, sample size, environmental influence, noise interference, and other factors, as well as to enhance the spectral characteristics [18]. The standard normal transformation is a relatively simple preprocessing method, which is often used in spectral preprocessing. Hence, we decided to use the standard normal transformation for spectral preprocessing as shown in Eqs. 2 and 3:

2 $$x_{snv,i} = \frac{{x_{i} - \overline{x} }}{{\sqrt {\frac{{\sum\nolimits_{i = 1}^{m} {\left( {x_{i} - \overline{x} } \right)^{2} } }}{m - 1}} }}$$

3 $$\overline{x} = \frac{{\sum\nolimits_{i = 1}^{m} {x_{i} } }}{m}$$

where, *m* represents the number of wavelengths.

#### Determination of the characteristic wavelength

Hyperspectral data contain a large amount of redundant information and information concerning collinearity. Characteristic wavelength screening should be performed before quantitative analysis to eliminate the influence of redundant information and improve the accuracy and stability of modeling [19]. SPSS v26 (IBM, Chicago, IL, USA) software was used to perform stepwise regression to screen characteristic wavelengths. This is one of the best predictive methods in multiple regression because it tests all independent variables and eliminates insignificant and meaningless variables by selecting valid independent variables that have a greater impact on the dependent variable in the equation. The use of this method ensures that the regression equation reasonably reflects the relationship between the independent variable and the dependent variable, making it suitable for use in this study [20].

#### Simulation acquisition method of different bandwidth data

**Fig. 2:**
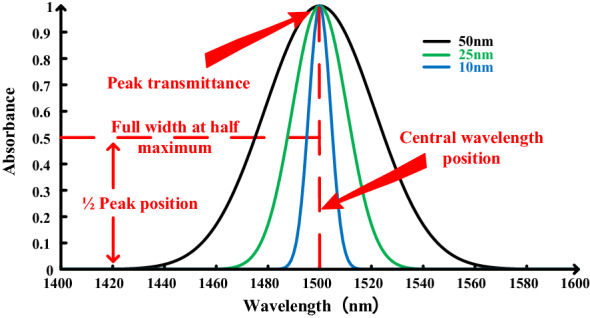
Filter transmission curve. The light transmittance curve when the center wavelength is 1500 nm, and the bandwidths are 10, 25, and 50 cm

Besides specifying the characteristic wavelength, determining the wavelength bandwidth is another key parameter for measuring nitrogen. If the spectrum used for modeling is too wide, it can lead to interference due to other factors when analyzing changes in absorbance. This in turn will reduce the prediction accuracy of the model [21]. On the contrary, it is easier to exclude a selected bandwidth. The narrower the modeled spectral range, the greater the accuracy of the model [22]. The bandwidth parameters of the existing filters on the market generally range from 10 to 300 nm. This experiment provides a basis for filter selection, as it compares the modeling effect under bandwidths of 10–300 nm through Matlab 2020a (The Math Works, Natick, MA, USA) simulation to determine the appropriate bandwidth range (Fig. 2).

The specific method is as follows:

The center wavelength and bandwidth parameters of each group were substituted into the approximate distribution function of light transmission of the filter to obtain the light transmission distribution curve corresponding to each group of wavelengths and bandwidths. Next, the convolution operation was performed on the hyperspectral data and distribution curve at the corresponding wavelength. The simulated data were obtained in line with the actual light transmission characteristics of the filter of each group of center wavelengths and bandwidths. Equations 4 and 5 demonstrate the approximate distribution function of light transmission of the band-pass filter [23]:

4 $$T(\uplambda ) = T_{p} \exp \left( { - \frac{{({\uplambda - \lambda }_{c} )^{2} }}{{2\sigma^{2} }}} \right)$$

5 $$\upsigma \,{ = }\,{{{\text{FWHM}}} \mathord{\left/ {\vphantom {{{\text{FWHM}}} {\sqrt {{2}\ln {2}} }}} \right. \kern-\nulldelimiterspace} {\sqrt {{2}\ln {2}} }}$$

where, *T*_*p*_ represents the peak transmittance of the filter, ideally *T*_*p*_ = 1; *λ*_*c*_ represents the center wavelength of the peak transmittance; and σ represents the variance of the waveform and has a linear relationship with the bandwidth (full width at half maximum, FWHM). The convolution calculation was performed as follows:

6 $$S_{i} { = }\sum\limits_{{T(\lambda ){ = 0}}}^{{1}} {A_{i} * } T(\lambda )$$

where, *S*_*i*_ represents simulation data and *A*_*i*_ represents the hyperspectral data (absorbance or reflectance value) at the corresponding wavelength.

#### Simulation method of obtaining the center wavelength shift data

The band-pass filter installed in this study is a thin-film interference filter. An increase in the temperature of the filter causes a change in the refractive index of the film. The substrate and the film have different coefficients of thermal expansion; hence, when heated, the film is elastically deformed under the action of substrate stress, resulting in a change in the concentration density and a shift in the center wavelength [24]. If the center wavelength shift due to thermal expansion is too large, the prediction performance of the model will be greatly reduced. In general, the center wavelength shift caused by the temperature effect is less than 10^–3^ nm °C [25]. To determine if the model built after installing the filter was affected by the ambient temperature, in this study, we used a model with a fixed center wavelength and bandwidth. The model predicted and analyzed the error situation of different offset data, and determined the ambient operating temperature range of the hyperspectral imaging detection device. The specific method was as follows:

Linear interpolation calculations were performed using the "spline" function in MATLAB 2020a based on the hyperspectral data with a high wavelength resolution of fresh corn leaves to obtain higher wavelength resolution data. The interpolation method is shown in Eq. 7.

The light transmittance distribution curve was obtained under the corresponding wavelength and bandwidth by substituting the wavelength shift from the center wavelength into the approximate distribution function of the light transmission of the filter.

The hyperspectral data and light distribution curve were convolved at the corresponding wavelength to obtain simulation data that conformed to the actual offset characteristics of the filter. For the convolution calculation, please see Eq. 6.

7 $$y = y_{i} + \frac{{x - x_{i} }}{{x_{i + 1} - x{}_{i}}}(y_{i + 1} - y_{i} )$$

For (*x*_*i*_, *x*_*i*+1_), any wavelength in the range *x*, the absorbance *y* can be calculated with Eq. 7. Among the variables, *x*_*i*_ and *y*_*i*_ represent the wavelength and absorbance, respectively, and *x*_*i*+1_ and *y*_*i*+1_ are the wavelength and absorbance, respectively, corresponding to the latter wavelength.

#### Evaluation indicators of the model

In this study, the accuracy of the model was evaluated using the determination coefficient (R^2^) and root mean squared error (RMSE) [26]. R^2^ is an indicator of the degree of fit of the model, and it ranges from 0 to 1 [27]. RMSE is a quantitative trade-off method and a common evaluation index, which can range from zero to infinity. The lower the RMSE value, the better the result [28]. The equations for R^2^ and RMSE are as follows:

8 $$R^{2} = \frac{SSR}{{SST}} = 1 - \frac{SSE}{{SST}}$$

where, *SST* represents the total sum of squares, *SSR* represents the regression sum of squares, and *SSE* represents the residual error sum of squares.

9 $$RMSE = \sqrt {\frac{1}{m}\sum\nolimits_{i = 1}^{m} {(y_{i} - \hat{y}_{i} )^{2} } }$$

where, *m* represents the number of samples; *y*_*i*_ represents true value.

## Results

### Selection of filter center wavelength

The model was simulated using the near-infrared hyperspectral data of 60 groups of fresh corn leaves in different growth stages. The stepwise regression method was combined with the existing filter models to process the hyperspectral data of the leaves. A filter with a characteristic wavelength of 1500 nm was selected. An increase in the full spectrum can reduce system error and eliminate background noise; therefore, in this study, we used a combination of 1500 nm and the full spectrum to conduct stepwise regression for modeling analysis.

In contrast to other experiments involving leaf water measurements, in this study, 60 randomly collected samples in different growth stages were divided into the modeling set and prediction set at a ratio of 3:1. The R^2^ and RMSE of the two models are detailed in Table 1; the results revealed that the water and the nitrogen modeling set and prediction set reached reasonable levels. According to the absorption characteristics of the near-infrared group, the frequency doubling absorption of the CH group is mainly contained around 1150 nm. Furthermore, the frequency doubling and the combined frequency absorption of the OH and CH groups are mainly contained around 1400 nm. When the characteristic wavelengths of the OH and CH groups are selected to establish a regression model, the absorption information of the CH groups negate each other. This indicates the rationale for selecting the two characteristic wavelengths for water measurements. However, a wavelength of 1500 nm only contains the NH2 group; hence, there is no need to consider interference due to other groups. At the same time, the double frequency absorption and combined frequency absorption of the N–H bond stretching vibration are around 1500 nm, which is also the absorption wavelength of the NH2 group. Therefore, it was reasonable to individually select the characteristic wavelength of 1500 nm as a characteristic of leaf nitrogen.

**Table 1:** Results of modeling and prediction based on feature wavelengths

Model	Wavelength (nm)	Calibration set		Prediction set	
		R^2^	RMSE	R^2^	RMSE
Nitrogen	1500	0.751	0.223	0.759	0.206
Water	1150, 1400	0.965	1.301	0.954	1.394

### Selection of filter center wavelength bandwidth

The parameters were substituted under the characteristic wavelength and different bandwidths into Eq. 4 (approximate light transmission distribution function) and the light transmittance distribution curve under the corresponding wavelength and bandwidth was obtained. Using Eq. 6, the hyperspectral data at the corresponding wavelength and the distribution curve were convolved, and the simulation data that accorded with the actual light transmission characteristics of the filter under different center wavelengths and bandwidths were obtained. The simulation data were applied under different parameters to establish a multivariate linear regression model. The following is a comparison of the modeling results of water and nitrogen under different bandwidths.

When the bandwidth was less than 50 nm, a significant change in R^2^ and RMSE of the model was not observed (Table 2). When the bandwidth was greater than 50 nm, the R^2^ and RMSE of the model were significantly reduced. When the bandwidth was less than 50 nm, the RMSE of the built model predicted that water content and nitrogen content were within the allowable error range, which can be used for general agricultural analysis. We finally selected 1500 nm as the characteristic wavelength, and building a nitrogen content model with a bandwidth of 25 nm, can accurately detect nitrogen in corn leaves.

**Table 2:** Modeling results for different bandwidths

Model	Water		Nitrogen	
	R^2^	RMSE	R^2^	RMSE
10	0.961	1.343	0.751	0.262
25	0.966	1.253	0.750	0.263
50	0.964	1.548	0.690	0.292
100	0.961	1.335	0.391	0.410

### Influence of the center wavelength shift on model prediction performance

We applied the interpolation formula 7 to obtain the wavelength data with a wavelength resolution, and included the shifted center wavelength and bandwidth (25 nm) in Eq. 4. The offset was set to obtain the light transmittance distribution curve after the center wavelength was shifted. Furthermore, the hyperspectral interpolation data of the corresponding wavelength were applied to Eq. 6 for convolution operation, and the simulation data conforming to the actual offset characteristic of the filter were obtained. We detailed the application of the model using a center wavelength (λ_c_) of 1500 nm and a bandwidth of 25 nm; the results of prediction detailing at different offset data are shown (Table 3).

**Table 3:** Effects of central wavelength drift on model prediction errors

Drift (nm)	RMSE	
	Nitrogen (g)	Water (%)
0.000	0.263	1.253
0.010	0.263	1.255
0.030	0.263	1.267
0.050	0.263	1.292
0.070	0.264	1.327
0.090	0.265	1.374
0.100	0.265	1.400
0.200	0.273	1.769
0.300	0.285	2.254
0.400	0.302	2.790
0.500	0.321	3.368

As shown in Table 3, the prediction error of the model increases with an increase in the center wavelength shift. A larger shift in the center wavelength led to a significant drop in the reliability of the model; however, the center wavelength shift of the filter was not greatly affected by temperature. Generally, the outdoor environment temperature varies within 50 °C, which is equivalent to a center wavelength shift within 0.05 nm, that is, the model prediction error was negligible.

### Construction of a near infrared detection device

**Fig. 3:**
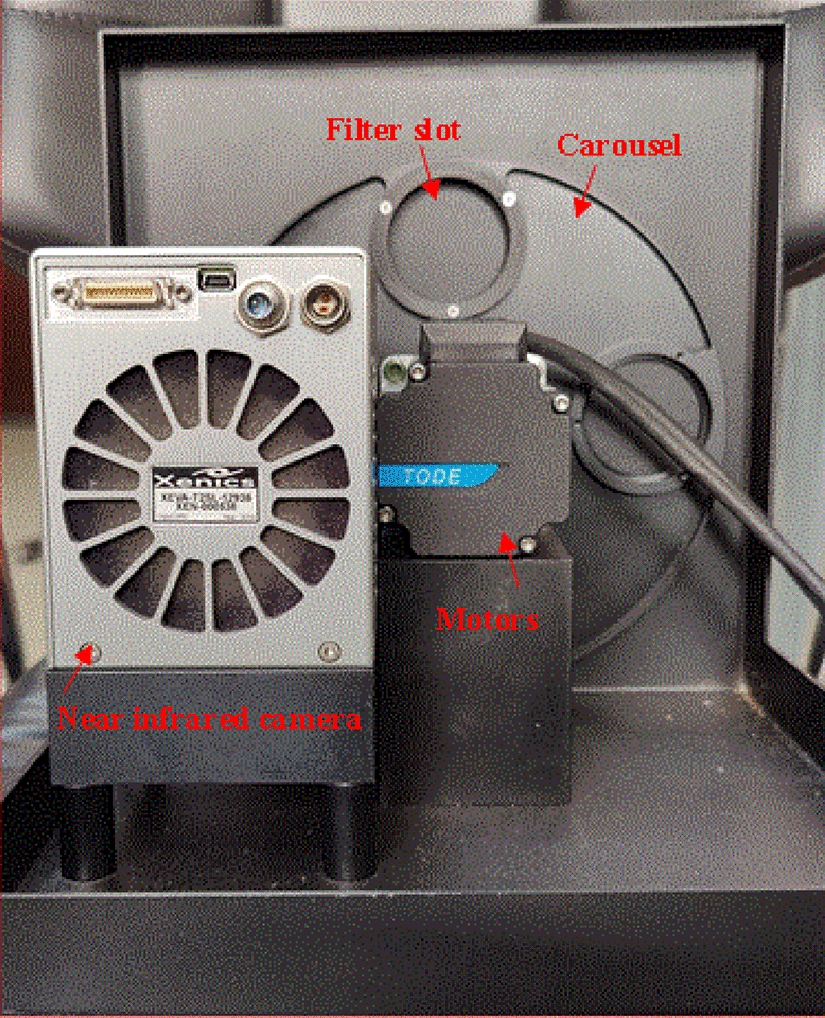
Near-infrared imaging device. The near-infrared imaging device built by filter splitting, with a high-sensitivity near-infrared camera, filter wheel, and servo motor, in which the near-infrared camera, servo motor, and filter wheel are placed inside the packaging box. The through holes of the camera, the packaging box, and the through holes in the runner are located on the same straight line

We applied theoretical and simulation analyses to obtain key parameters such as the location and number of characteristic wavelengths, bandwidth, and offset limits of the characteristic wavelengths required for leaf water measurement. Based on the data obtained using the Xanic XEVA-2.35 near-infrared camera, a near infrared detection device was built that was suitable for in-situ detection of live leaves in the field (Fig. 3).

**Fig. 4:**
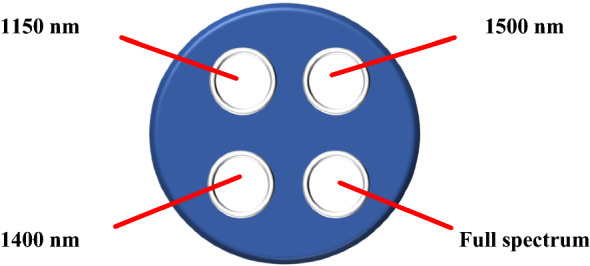
Filter holder. According to the actual size of the filter, the filter wheel is designed to install four filter wheels of diameter 50 mm, and the filter is embedded in a 52-mm plastic ring to fix it

According to the actual size of the filter, a filter wheel was designed to install four filters of diameter 50 mm (Fig. 4). When it was necessary to use a filter, which corresponds to a certain wavelength, the rotating shaft of the servo motor caused the slot in the middle of the filter wheel to rotate to the desired position. The design of the filter wheel helps avoid inaccurate measurements caused by the contamination of the filter lens due to improper manual operation. At the same time, to prevent light leakage caused by a poor fit between the filter wheel and camera lens, we designed a corresponding mechanical system to solve the Newton ring phenomenon that must be faced in real-time inspection in the field.

### Predicting fresh corn leaf water content using the near-infrared detection imaging device

The determination coefficient of leaf water content predicted using the near-infrared imaging device reached 0.930, and the root mean square error reached 1.030%, which is consistent with the results obtained by hyperspectral simulation, indicating that the near-infrared imaging device has a good effect on quantifying corn leaf water (Table 4).

**Table 4:** Results of modeling based on different filters

Wavelength (nm)	R^2^	RMSE
Full spectrum	0.437	2.940
1150	0.362	3.120
1400	0.597	2.480
1400, Full spectrum	0.922	1.090
1150, 1400, Full spectrum	0.930	1.030

This study provides support for a new method of in-situ detection using hyperspectral image technology to visually analyze the internal chemical components of live corn leaves.

**Fig. 5:**
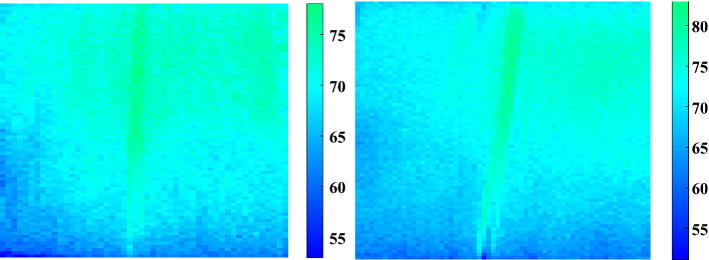
Near-infrared imaging device predicts the results of in-situ imaging detection of water content in live maize leaves. Predicted distribution of corn leaf water content obtained using the near-infrared imaging device. The image on the left is water content distribution in the extraction area of the leaves, and the color bar on the right is water content distribution. The colors and shades of different positions on the leaves indicate the corresponding water content values at that position. According to the distribution map, the predicted value of the water content of each pixel can be obtained

The main leaf veins of the living leaf in the figure were not destroyed and imaging results in the case of transpiration tension transported water indicated that the main leaf veins had a higher water content (Fig. 5). From the visual imaging of the leaf structure, it was observed that the leaf loses water from the edge, which gradually spreads to the middle of the leaf. The water content in the leaf petiole was significantly higher than that in the mesophyll tissue. The imaging results were consistent with the actual situation. Furthermore, the difference in the gradient value of water content in different areas of the leaf veins and mesophyll was significantly greater than the actual detection accuracy value (compared with the drying method, the average error was approximately 1.5%). Accurate and reliable water imaging detection results and their analysis can indicate that the application of average water content of leaf veins and mesophyll tissue and their differences, and their dynamic difference values, are suitable for characterizing the physiological indicators of drought resistance identification.

**Fig. 6:**
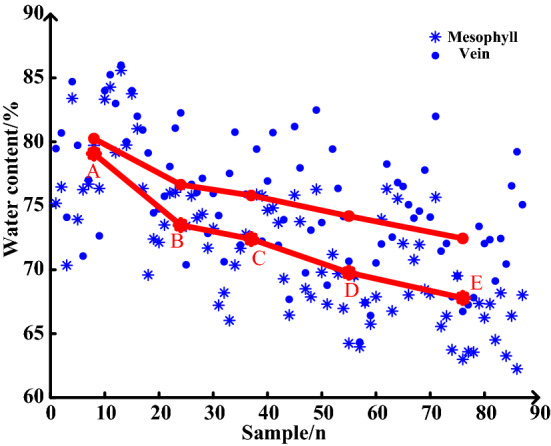
Maize leaf water in different growth stages. The red dots represent the average values of different growth stages. After shooting the leaves, the mesophyll and vein tissue were separated, and the water content was measured using the drying method

The average values of water content were measured at different reproductive stages, revealing a higher level in veins than that in the mesophyll tissue. Further, as the leaves grew, there was an increase in accumulated organic matter; hence, a lower water content was observed with regards to leaf proportion, which indicates a trend of decreasing water content (Fig. 6).

The water content in maize leaves predicted by the near infrared detection device was consistent with the physiological biochemistry of the crop, further indicating that the model constructed from the spectral data can predict leaf water content. In the subsequent studies, we will establish and validate the models of the relationships between WV, WM, DVM; their dynamic changes; leaf water potential; relative water content; and transpiration rate characterization to achieve the in-situ detection of physiological indicators of drought resistance in vivo in crop leaves. Furthermore, a significant amount of data concerning WV, WM, DVM, ∆WV, ∆WM, ∆DVM, and biomass index of maize under different drought stress intensities, different fertility periods, and different diurnal periods were directly applied to conduct a comprehensive index study to characterize the drought resistance of the crop.

### Predicting fresh corn leaf nitrogen content using the near-infrared detection imaging device

The nitrogen content in the leaves was obtained by simulation using hyperspectral data, the coefficient of determination reached 0.750, and the root mean square error reached 0.263 g (Table 3).

**Fig. 7:**
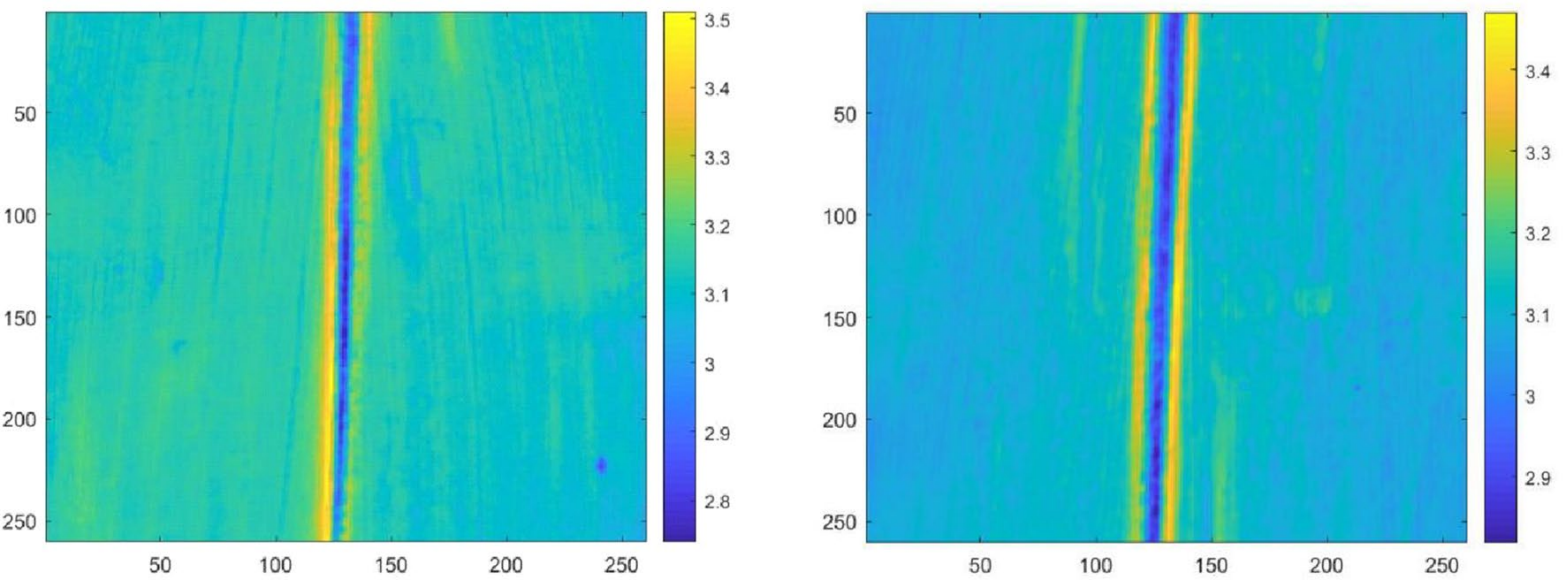
Prediction of fresh maize leaf nitrogen content imaging assay results. The nitrogen content in the leaves was mapped using high-precision model detection results, visual imaging analysis of corn leaf nitrogen, extracting the absorbance value of each pixel in each image at the characteristic wavelength of 1500 nm, and calculating the nitrogen content of each pixel of the corn leaf by the regression model

Hyperspectral data and models were applied to predict the results of imaging the nitrogen content of maize leaves (Fig. 7). The nitrogen content of the main leaf vein is significantly lower, and the nitrogen content of the mesophyll area close to the main leaf vein is significantly higher. The distribution characteristics and patterns of leaf nitrogen content obtained by imaging are consistent with the actual distribution of maize leaves. The results indicate that non-destructive and real-time imaging detection of the nutritional status of maize can be performed using spectral characteristics, which can provide a novel and effective technical means to study the storage and transportation of nitrogen in live crops. The simulated "low-resolution, high flux" spectrum can meet the requirements for the detection and imaging of the average nitrogen content as well as the nitrogen content in different regions of the leaf.

## Discussion

Effective and rapid identification of crop drought resistance is important to improve the breeding efficiency of crop tolerance under abiotic stress such as drought. Furthermore, it will aid in understanding the mechanism of drought resistance in crops. Current research shows that leaf water potential, relative water content, transpiration rate, and stomatal conductivity are closely related to drought resistance [29]. Among these, there is a lack of rapid in vivo detection methods for leaf water potential and relative water content that can be conducted in-situ. The application of near-infrared hyperspectral imaging could distinguish large differences in the water content in leaf veins and fleshy regions of fresh maize leaves [30]. Through the combination of leaf water potential, relative water content, and transpiration rate and analysis, it is feasible to use the average water content of leaf veins and mesophyll tissue and their differences (WV, WM, and DVM), and their dynamic difference values (∆WV, ∆WM, and ∆DVM), to characterize leaf water potential, relative water content, and transpiration rate, respectively. Three of the four important physiological indicators of drought resistance identification can be achieved. Furthermore, by measuring stomatal conductance, which is closely related to the transpiration rate, all four important physiological indexes for drought resistance can be determined. Due to the difficulties in using large-volume and heavy-weight hyperspectral instruments for the in-situ detection of live crop leaves in the field, a smaller NIRDR-AI device was constructed for this study that was suitable for the in-situ detection of live leaves in the field, which was used to visually image the water inside leaves.

**Fig. 8:**
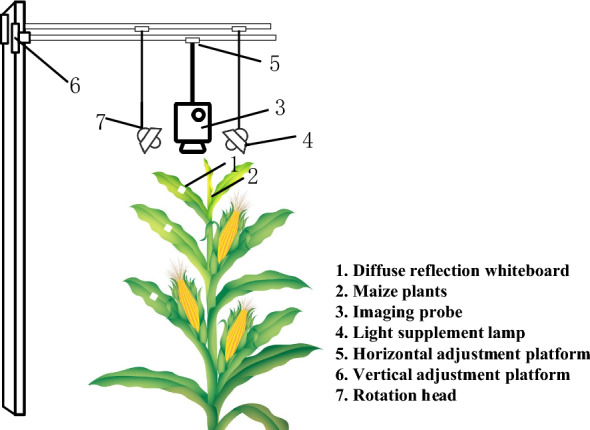
Working principle of near-infrared imaging device. The near-infrared imaging device can be used to image and analyze the leaves of a single corn plant, in which important parameters of the light path can be automatically adjusted

Currently, we are conducting experiments under field conditions. The working principle of the NIRDR-AI device is that the upper computer controls the motor to rotate the filter wheel to an appropriate angle and collect information concerning the leaves. The quantitative analysis of the water and nitrogen content is completed by converting the collected near-infrared images at specific wavelengths into near-infrared spectra (Fig. 8). We used horizontal and vertical adjustment devices to adjust the near-infrared camera to an appropriate position and we added fill-light in accordance with factors such as weather, and placed a whiteboard on the blade for calibration when recording.

This experiment was under taken with a near-infrared imaging device under fill light conditions indoors. Subsequently, investigations on whether it is feasible to use sunlight in the field to shoot were carried out (Table 5). Pixel intensity of the photo reaches the maximum gray value of 16,384 under sunlight, which indicates it can achieve saturation, similar to in the fill light condition. In addition, it demonstrates that it is feasible to directly use sunlight in field experiments.

**Table 5:** Modeling results for different bandwidths

Model	Maximum gray value under different filters (Adu)			
	1150	1400	1500	Full spectrum
Fill light	16,384	16,384	16,384	16,384
Sunshine	16,384	16,384	16,384	16,384

Some limitations associated with the experiments are as follows. The camera lens selected in this experiment is a short-focus lens, which has a small focal length and a wide viewing angle. Therefore, it is more suitable for quantitative analysis of canopy leaf water. Telephoto lenses will be used in subsequent experiments, which would yield stronger scenes, increase pixel occupancy, and more accurate modeling results; furthermore, the results of visual analysis of leaf stalk and mesophyll tissue of a single leaf would be more detailed.

## Conclusions

In this study, we aimed to obtain key technical parameters of a multispectral imaging detection device suitable for real-time measurement of water and nitrogen content in live maize leaves in the field using hyperspectral data simulation. We set up the device according to the selected key parameters. Using this device, basic testing was achieved and in-situ imaging detection of water content and nitrogen content in live corn leaves in a field environment was completed. The proposed indicators and methods, such as WV, WM, DVM, ∆WV, ∆WM, and ∆DVM, could facilitate the elucidation of drought resistance mechanisms in crops. The near infrared detection device built in this study has wider research and application potential, such as in-situ imaging of in vivo leaf chlorophyll and cellulose chemistry in a field environment. The device introduced in this article could not only provide a novel approach for the identification of drought resistance in crops but could also be used for crop nutrient status assessment, with a wide range of potential applications.

## Data Availability

Not applicable.

## Abbreviations

FWHM: Full width at half maximum
NIRDR-AI: Near-infrared diffuse reflection agile imaging
NIR-R: Near-infrared red
NIR-RE: Near-infrared red-edge
RMSE: Root mean squared error
